# New Digital Plug and Imaging Sensor for a Proton Therapy Monitoring System Based on Positron Emission Tomography

**DOI:** 10.3390/s18093006

**Published:** 2018-09-08

**Authors:** Nicola D’Ascenzo, Min Gao, Emanuele Antonecchia, Paolo Gnudi, Hsien-Hsin Chen, Fang-Hsin Chen, Ji-Hong Hong, Ing-Tsung Hsiao, Tzu-Chen Yen, Weidong Wang, Daoming Xi, Bo Zhang, Qingguo Xie

**Affiliations:** 1 School of life science and technology, Huazhong University of Science and Technology, Wuhan 430074, China; april_mingao1@hust.edu.cn (M.G.); emanuele.antonecchia@neuromed.it (E.A.); 2 Wuhan National Laboratory of Optoelectronics, Wuhan 430074, China; 3 Istituto Neurologico Mediterraneo, NEUROMED IRCCS, Pozzilli 86077, Italy; Paolo.Gnudi@student.hs-rm.de; 4 Department of Biomedical Engineering, RheinMain University of Applied Sciences, Wiesbaden 65203, Germany; 5 Department of Medical Imaging and Radiological Science, Chang Gung University, Taoyuan 33302, China; hsin0426@gmail.com (H.-H.C.); fanghsinchen@mail.cgu.edu.tw (F.-H.C.); ihsiao@mail.cgu.edu.tw (I.-T.H.); 6 Department of Radiation Oncology, Chang Gung Memorial Hospital at Linkou (CGMH at Linkou), Taoyuan 33302, China; jihong@cgmh.org.tw; 7 Molecular Imaging Center and Nuclear Medicine, Chang Gung Memorial Hospital/Chang Gung University (CGMH/CGU), Taoyuan 33302, China; yen1110@cloud.cgmh.org.tw; 8 Institute for Radiological Research, Chang Gung Memorial Hospital/Chang Gung University (CGMH/CGU), Taoyuan 33302, China; 9 Key Laboratory of Biomedical Engineering and Translational Medicine, Ministry of Industry and Information Technology, Beijing 100191, China; wangwd301@126.com; 10 Raycan Technology Co., Ltd., Suzhou 215163, China; daoming.xi@raymeasure.com; 11 RaySolution Digital Medical Imaging Co., Ltd., Ezhou 436044, China; bo.zhang@digital-pet.com

**Keywords:** silicon sensors, digital readout, multi voltage threshold, positron emission tomography, proton therapy

## Abstract

One of the most challenging areas of sensor development for nuclear medicine is the design of proton therapy monitoring systems. Sensors are operated in a high detection rate regime in beam-on conditions. We realized a prototype of a monitoring system for proton therapy based on the technique of positron emission tomography. We used the Plug and Imaging (P&I) technology in this application. This sensing system includes LYSO/silicon photomultiplier (SiPM) detection elements, fast digital multi voltage threshold (MVT) readout electronics and dedicated image reconstruction algorithms. In this paper, we show that the P&I sensor system has a uniform response and is controllable in the experimental conditions of the proton therapy room. The prototype of PET monitoring device based on the P&I sensor system has an intrinsic experimental spatial resolution of approximately 3 mm (FWHM), obtained operating the prototype both during the beam irradiation and right after it. The count-rate performance of the P&I sensor approaches 5 Mcps and allows the collection of relevant statistics for the nuclide analysis. The measurement of both the half life and the relative abundance of the positron emitters generated in the target volume through irradiation of $10^{10}$ protons in approximately 15 s is performed with 0.5% and 5% accuracy, respectively.

## 1. Introduction

Optical sensing imaging systems in nuclear medicine are based on the principle of conversion of the energy of gamma rays into visible light in scintillation materials. Optical sensors are used to read out the scintillation light and to convert it into an electrical signal for further analysis. The typical wavelength sensitivity of modern sensors in this application should peak between 400 nm and 420 nm because the relevant scintillators emit light in this wavelength range. Monitoring systems for the dose delivered during proton therapy treatments are among the most critical applications in nuclear medicine. Recent advances in this area require in fact to design and test new image sensor systems with high rate detection capability in the wavelength range mentioned above. The development of dedicated digital readout electronics, possibly integrated within the sensor, represents a key issue in this respect [1,2].

Positron emission tomography (PET) has been demonstrated to be the most efficient technique to control the delivered dose during proton therapy treatment in the 1990s [3,4,5,6]. Building on the study of the distribution of the positron-emitting nuclei generated by relativistic ion beams in biological matter, the first PET systems were installed in proton therapy treatment rooms. Since then, PET is dominating the panorama of modern research in the field of novel sensor development for frontier applications in nuclear medicine and radiation therapy, with the unique synergy of a large number of groups [7,8,9,10,11,12,13,14,15,16,17,18,19,20,21,22,23,24,25,26,27]. During a proton therapy treatment, cancer cells are bombarded with protons at energies typically ranging between 70 MeV and 250 MeV. As ions are entirely stopped in the target volume, a reliable monitoring of the delivered dose can rely only on the detection of secondary radiation. PET-based proton therapy monitoring systems target the non-elastic nuclear interactions of protons with the target volume. Positron emitters as ^11^C, ^10^C, ^15^O, ^13^N, ^12^N and ^8^B are generated with abundance ranging between 0.1% and 70%. The time-correlated 511 keV photon pairs produced in the annihilation of the emitted positrons are detected. Tomographic image reconstruction techniques are applied to the data to retrieve the space distribution of the positron emitters within the target volume during and after proton irradiation.

Information about the precision of the treatment is obtained by comparing the measured positron distribution in the target volume with a prediction based on the treatment plan and on the time course of the irradiation [28]. Recent improvements in the physical modeling of nuclear interactions in biological matter allow monitoring the maximum ion range, verifying the field position and quantifying the unpredictable deviations between prescribed and applied dose due to biological and anatomical changes in the patient [29].

Optical sensing for PET imaging plays a fundamental role in the design of highly accurate monitoring systems for proton therapy. Scintillating crystals read out by photomultiplier tubes-related technology constitute a traditional sensor solution for the detection of 511 keV photons. By way of example, a prototype of dual panel PET detector with area 15×15 cm^2^ was recently built based on a LYSO crystal matrix with 2 mm pitch read out by a position sensitive photomultiplier (H8500; HAMAMATSU) [30,31]. It was demonstrated that the presence of materials with different oxygen content can be detected with this technique. Although such technology allows a 1:1 coupling between crystals and photodetectors and is therefore suitable to the requirements of PET monitoring systems, it needs a bias voltage up to 1.5 kV and constrains the design to blocks with minimal size of approximately 5×5 cm^2^.

Besides traditional photodetectors, one of the most suitable sensors for this application is the silicon photomultiplier (SiPM). It is a low photon flux silicon sensor with an area ranging typically between 1 mm^2^ and 9 mm^2^ and an internal gain of $10^{6}$ reached at a bias ranging between 20 V and 60 V. Modern commercially available SiPMs exhibit photon detection efficiency up to 40% at 420 nm and single photon time resolution less than 100 ps (FWHM) [32,33,34]. Radiation hardness is currently under investigation [35]. Due to their compact size and simple required readout electronics, they are substituting the traditional photomultiplier tubes in PET instrumentation for proton therapy monitoring systems. LYSO crystals with size 3.8×3.8×22 mm^3^ and read out by SiPM (dSIPM; PHILIPS) were used to build a dual panel PET detector with area 6.5×6.5 cm^2^ [36]. To reduce the dark count rate (DCR) of the sensors, the system was kept at a temperature of 3 °C. The modules were placed in separate Styrofoam boxes, which were continuously flushed with dry nitrogen gas to prevent condensation. The total DCR was further reduced significantly by disabling the top 20% of the microcells that exhibited the highest DCR. This prototype allowed a measurement of the fast decay of ^12^N, which has an half-life of just 11 ms, during the proton irradiation, increasing the rejection of the prompt photon background and consequently improving the image quality. SiPMs (MPPC; HAMAMATSU) are also currently used in the prototype of the INSIDE PET scanner, for the readout of Lutetium Fine Silicate (LFS) crystals with size 3×3×20 mm^3^ composing the 10×25 cm^2^ PET panels [37,38].

As it follows from the above-mentioned detector concepts, the SiPM enabled to reach a new frontier in the sensor development for PET monitoring systems for proton therapy, namely the in-beam image acquisition. PET detectors need to be integrated with the proton delivery equipment and the signal registration is performed either during irradiation or immediately after it without moving the patient. The in-beam signal registration is beneficial to increment the collected signal statistics and to reduce the effect of biological washout, with a consequent improvement of the image quality and accuracy. The key issue is here the high level of background due to the prompt photons generated in nuclear interactions during proton irradiation. Several solutions are being tested, based on the background rejection using the spill time information [39]. Besides PET, other approaches to range determination using the prompt photons information were also developed [40,41,42,43,44,45,46,47,48,49,50].

In recent years, we have witnessed an improvement both in the development of dedicated SiPM technology based on the complementary metal oxide semiconductor (CMOS) process [51,52] and in the realization of related high bandwidth digital readout electronics for PET [53,54,55,56,57,58,59,60,61,62,63]. As a possible solution to the sampling-rate challenge, we have previously proposed a multi voltage threshold (MVT) sampling method that takes samples of a pulse with respect to a set of reference voltages. By choosing the reference voltages properly, the MVT method can always obtain samples at the fast leading edge of a pulse. The total energy and the timing of the pulse can be obtained by the digital signal processing of a small set of samples of the pulse. We have shown that for PET detectors it is sufficient to use only a few reference voltages to obtain a small number of samples. As the pulses are instantaneously digitized and the number of samples per pulse is small, this sampling method can have a high count-rate capability without requiring a large on-board storage buffer [64,65]. This fast digital electronics is used to read out dedicated sensors based on the LYSO/SiPM coupling. The sensors are assembled in a compact configuration, which enables the design of PET systems with geometries adaptable to specific organs or clinical problems. A dedicated geometry-independent image reconstruction framework has been developed in order to provide a fast and effective analysis of the data collected by this sensor system. The combination of novel LYSO/SiPM sensor technology, MVT dedicated digital electronics and image reconstruction constitutes the Plug and Imaging (P&I) sensor. We showed that, in optimal conditions, this imaging system can reach a crystal-level energy resolution of approximately 10% and a detector-level coincidence time resolution of 390 ps FWHM [56]. The system can support a maximal count rate of approximately 5 Mcps [62], being thus competitive with similar sensor and readout technologies for PET.

In the technological context outlined above, the P&I sensor system would satisfy the demanding requirements of proton therapy monitoring systems. However, while we have already demonstrated its possible application to both preclinical and clinical standard PET devices, its feasibility for non-conventional systems has not been investigated yet. In this paper, we show, for the first time to our knowledge, the application of the P&I sensor system to the more challenging scenario of proton therapy monitoring with PET. We built a prototype PET monitoring system on the basis of the P&I sensor modules and we installed it at the proton delivery facility of the Chang Gung Memorial Hospital. In this paper, we show that the P&I sensor system composed of LYSO/SiPM sensors with MVT dedicated digital readout and reconstruction has a uniform response and is controllable in the experimental conditions of the proton therapy room. The prototype of PET monitoring device based on the P&I sensor system has an intrinsic experimental spatial resolution of approximately 3 mm (FWHM). A key-feature of this paper is that the count rate performance of the P&I sensor approaches 5 Mcps and is higher than most of the reported prototypes of proton beam monitoring systems with digital readout. It allows both the beam-on operation and the collection of relevant statistics for a nuclide analysis in the beam-off time. We measure both the half life and relative abundance of the positron emitters generated in the target volume through the irradiation of $10^{10}$ protons in approximately 15 s with respective accuracies of 0.5% and 5%.

The paper is structured as follows. In Section 2, we present the P&I sensor technology, the PET monitoring system prototype and the research methods and purpose; in Section 3, we outline the results divided into measured technological parameters of the optical sensing system and prototype performance; and, finally, in Section 4, we discuss the results and perform a comparison with recent optical sensing systems developed for PET proton therapy monitoring devices.

## 2. Materials and Methods

### 2.1. The Plug and Imaging Sensor System

**Figure 1:**
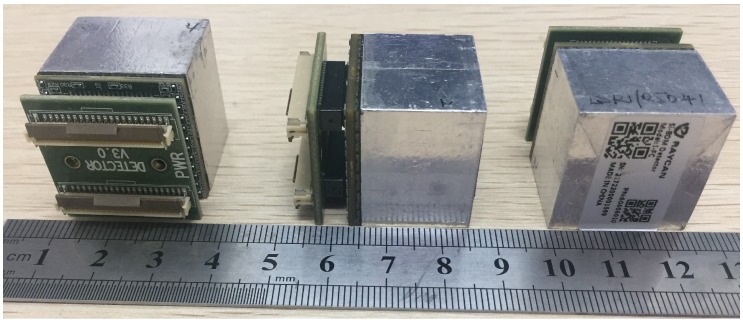
The P&I sensor system. A 6×6 array of LYSO crystals with size 3.9×3.9×20 mm^3^ is read out by an array of 6×6 SiPM. The assembled block has a dedicated plug-in system for the readout electronics on the back.

The basic module of the P&I imaging sensor system is shown in Figure 1. It is composed of a 6×6 LYSO array read out by a 6×6 SiPM array (FM30035; SENSL, Cork, Ireland) and a printed circuit board (PCB) with the SiPM biasing circuit. The used SiPM is composed of 4774 microcells with a pitch of 35 μm and a filling factor of 64% on a total area of 3×3 mm^2^. It exhibits a 20% peak photon detection efficiency at the wavelength of 500 nm. The average breakdown voltage is 27.5 V, with a temperature dependence of 20 mV/K. The LYSO matrix (JTC, Suzhou, China) contains 3.9×3.9×20 mm^3^ pixellated crystals with 0.3 mm crystal gaps that are filled with barium sulfate to reflect the scintillation light and isolate each crystal optically. Each SiPM has an active detection area of 3×3 mm^2^. The SiPM and LYSO matrices, both having a 4.2×4.2 mm pitch, are carefully aligned and coupled using epoxy optical adhesive (China Bluestar Chengrand Co., Ltd., Chengdu, China, Model: GHJ-01(Z), refractive index 1.56, thickness approximately 0.1 mm, light transmittance >90% for visible wavelengths, operation temperature –60 °C– 80 °C). LYSO/SiPM elements are read out independently. The overall detection area of the module is approximately 25×25 mm^2^.

**Figure 2:**
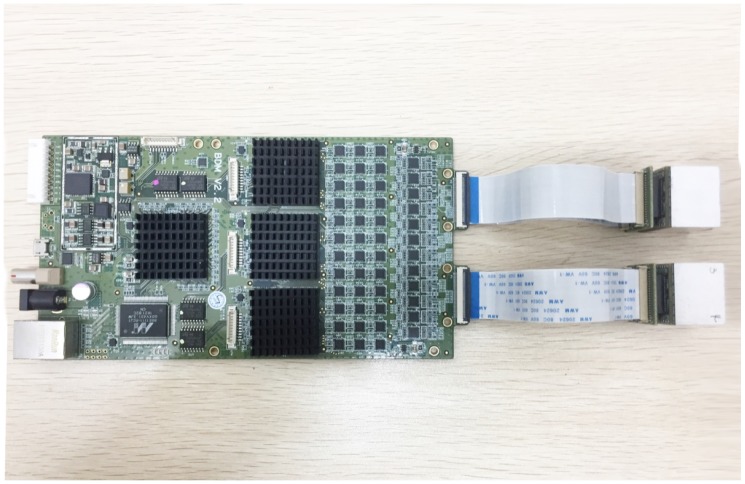
The P&I sensor system. Two P&I modules are connected to a MVT readout board.

**Figure 3:**
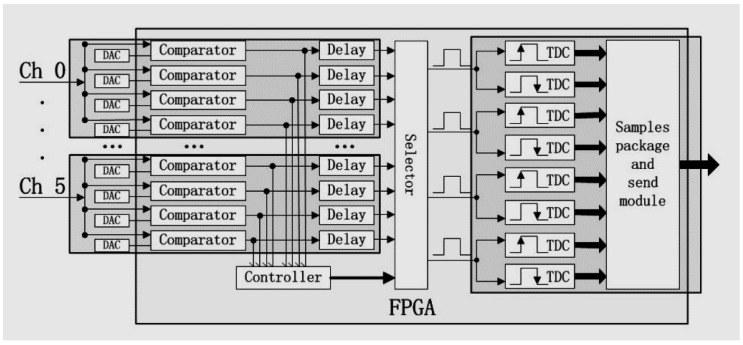
The P&I sensor system. Schematics of the digital readout electronics based on the MVT concept.

The digital readout electronics is shown in Figure 2. One electronic board serves two P&I sensor modules, providing thus the biasing and readout to 72 independent SiPM/LYSO channels. Concerning the biasing, due to the intrinsic wafer non-uniformities in the production of the SiPMs, the sensors do not have the same breakdown voltage. The SiPMs used in the prototype have an average breakdown voltage of 24.5 V, with a variation of 8% (FWHM). We grouped them so that each pair of P&I modules exhibits a variation of approximately 2% (FWHM). An average overvoltage of 5 V is provided to each pair of modules by a board. Concerning the readout, as shown in Figure 3, we designed a two-channel FPGA-only MVT-digitizer board by using an Altera EP4CE115F29I7N FPGA, with 114,480 logic elements (LE), 3888 Kbits embedded memory, 266 embedded 18×18 multipliers, 4 general-purpose PLLs, 20 Global Clock Networks, 8 User I/O banks, and 528 maximum user I/O. A 24-Channel 4-level MVT digitizer is using 51% LE and 46% I/O of the FPGA chip. We are currently developing a 36-ch 4-levels MVT digitizer using the same chip. Each MVT channel has four comparators to provide four reference voltages. A key feature of the implementation is to use the differential I/Os of the FPGA working in the low voltage differential signaling (LVDS) receiver mode to function as voltage comparators (they are referred to as the LVDS comparators below). A PET event pulse generated by a LYSO/SiPM sensor is split into four signals, each of which is sent to an LVDS comparator. The logic output of the LVDS comparator is connected to two TDCs for determining the digital times of its positive and negative transitions, therefore generating a total of eight samples for a pulse with sufficient amplitude. Since the voltage values are known a priori, these samples need to contain the time values generated by the TDCs. The eight time samples generated for a pulse are packaged into an event-word and stored in a FIFO. The FIFO data are sent out for analysis via an Ethernet interface. The LVDS comparators, TDCs, event identify and pack module, and FIFO are all implemented on the FPGA. In addition, a data send module for talking to an Ethernet interface is implemented in the FPGA. The TDC is governed by a 200 MHz clock and the counter (the digital coarse time) has 40 bits, capable of counting up to about 91.7 min at 5-ns increment. In other words, the time stamp of the TDC is composed of two parts, a coarse time and a fine time. The coarse time is obtained with the counter and has a resolution of 5 ns, the fine part is limited by the 90 ps minimal bin width of the TDC. The reference voltages are provided using Texas Instrument (TI) DAC7678. All MVT channels are synchronized to an external sync clock. With respect to the previous implementation [56], a 1 G Ethernet interface is used. Each threshold produces two 48-bit time samples that are packed into a 192-bit record. Therefore, a maximal count rate of 5 Mcps is achievable. The design is compatible with a 10 G Ethernet interface, which would further improve the maximal count rate allowed.

The intrinsic properties of the LYSO/SiPM sensor impose a physical limitation to the count rate per channel. As the decay time of the LYSO crystal is approximately 40 ns, the whole generated signal has a duration of less than 200 ns. By using a paralizable dead-time model with a 200-ns dead time, we have <10% dead-time loss at event rates below approximately 160 kcps per channel.

### 2.2. The Prototype of P&I PET for Proton Therapy Monitoring

**Figure 4:**
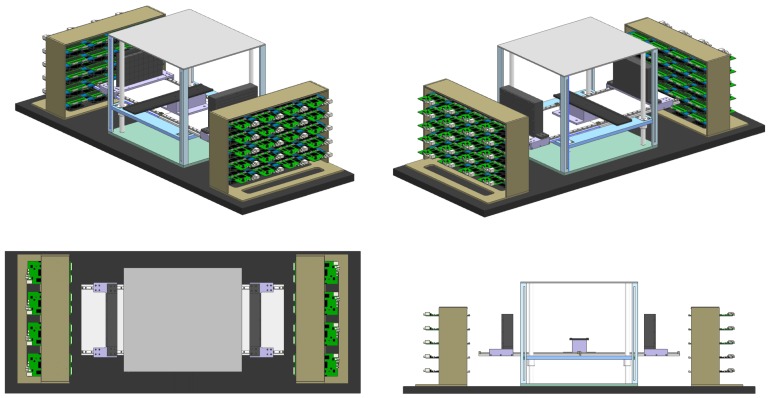
Different views of the design of the prototype of P&I PET monitoring system for proton beam therapy. The detector modules (flat black panels), the electronic board housing (side structures) and the central positioning support for the alignment of phantoms and detectors are placed on a movable base adaptable to the proton therapy room and fixable to the proton therapy patient’s bed.

The P&I sensor modules described in the previous section were used to design a prototype PET monitoring system for proton therapy. Different views of the design are shown in Figure 4. The prototype consists of two flat panel PET heads composed of a 8×5 P&I modules. The total area of each module is approximately 20×12.5 cm^2^. The total number of LYSO/SiPM channels is 2880. The distance between the modules is adjustable and is set to 20 cm in this study. A central support is used to align the target volume with the detector system and place it at the center of the field of view (FOV). As the system is composed of 80 P&I heads, 40 MVT electronic boards are needed for the readout. A housing support for the boards is placed at both sides of the prototype. A fan cooling system is used to keep the temperature of the prototype stable. Temperature close to the chip is constantly measured, but we did not include a temperature feedback control for the system settings. The whole structure is mounted on a proton therapy patient bed, which can be fixed to the movable stage of the proton therapy room.

**Figure 5:**
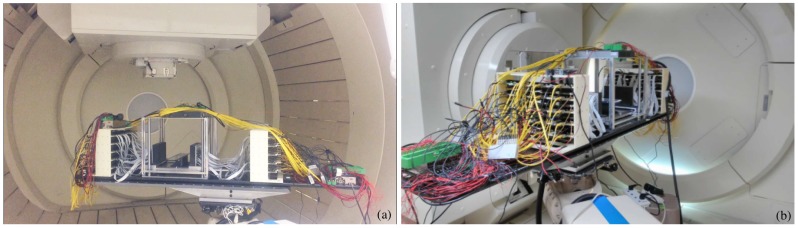
Installation of the prototype of Plug and Imaging sensor PET inside the proton therapy treatment room at the proton therapy center of the Chang Gung Memorial Hospital. The prototype is shown: during the table rotation (**a**); and in the final position, aligned with the proton beam (**b**).

The prototype was installed in the proton beam treatment room, aligned with the proton beam delivery system, at the proton therapy center of the Chang Gung Memorial Hospital of Taipei. A cyclotron generates static 230 MeV proton beams for clinical radiotherapy. An energy degrader and a series of beam instrumentations restrict the beam size and the energy range. After passing through a magnet beam transport system, the beam is delivered with submillimetric precision in the proton therapy room with a rotating gantry base with a diameter of 10.6 m. The installation of the prototype in the treatment room is shown in Figure 5. The prototype was placed on the same robotic coach system used for patients (Figure 5a) and resulted in an alignment with an accuracy of 0.01 mm after the gantry rotation (Figure 5b).

### 2.3. Phantom Irradiation

The experiment followed the ethical guidelines and was approved by the Chang Gung Memorial Hospital of Taipei. We prepared a water-filled phantom and a PMMA phantom with density 1.18 g/cm^3^ and relative composition of 8.06% hydrogen, 59.98% carbon and 31.96% Oxygen (weight percentages). The phantoms had a transverse section of 5×5 cm^2^ and a length of 20 cm along the beam direction. They were irradiated with a 150 MeV pencil beam aligned with its center. The beam had a Gaussian transversal profile with full width half maximum of approximately 2 mm and 0.3% energy spread.

The irradiation of the water phantom was performed with high proton statistics of approximately 10^11^ protons corresponding to a total dose of approximately 8 $\times \;10^{3}$ cGy delivered within a time of approximately 180 s. The irradiation of the PMMA phantom was performed with high proton statistics of approximately 10^10^ protons corresponding to a total dose of approximately 800 cGy delivered within a time of approximately 15 s.

Data were acquired during the beam-on time and during the following 3600 s after the beam is turned off (beam-off period). During the beam-on time, we reached a saturated count rate of 5.2 Mcps. When the beam was off, the count rate decreased to approximately 3.2 Mcps.

Before and after each irradiation, a system calibration run was performed with a phantom containing two ^22^Na point-like sources placed in the middle plane of the system. The first one was placed in the center of the FOV, and the second one at a distance of 5 cm along the beam direction. A total statistics of 10^5^ hits per channel was collected in 10 min acquisition and was used to verify the operational condition of the system.

### 2.4. Data Acquisition and Processing

The four voltage thresholds needed by the MVT digital readout electronics were set to 20 mV, 115 mV, 210 mV and 305 mV during the acquisition. These settings were tuned to guarantee a time resolution and an energy resolution of approximately 20% and 500 ps [56]. If a signal in a channel crosses the four thresholds, the eight crossing times measured by the TDC are stored in a raw data file, which contains all acquired hits without any selection. The data processing was performed offline after acquisition.

The crossing time at the 20 mV threshold was taken as time stamp of the hit and was expressed in ns. The eight samples were fit with the function:

(1) $$V\left(t\right)=a\exp\left(-bt\right)\left(1-\exp\left(-ct\right)\right)$$

The integral of the fitted function within a time window of 200 ns was taken as the energy of the hit and was expressed in arbitrary units (AU).

The data of calibration runs obtained with the ^22^Na sources were used to identify for each channel the position and the width of the photoelectric peak corresponding to the 511 keV gamma ray. The energy spectrum of each channel was analyzed. The mean value and the width of the photoelectric peak were estimated with a gaussian fit and the values were stored in a look up table.

The calibration of the channels was used to further process the collected data after phantom irradiation. Singles were formed imposing to the reconstructed hits a symmetric energy window with a total width of 2 σ around the tabulated value of the photoelectric peak. The position of the singles was taken as the position of the center of the LYSO crystal of the corresponding channel. Finally, coincidences were sorted out of the singles imposing a coincidence time window of 10 ns for the beam off data and 5 ns for the beam-on acquisition. The shorter coincidence time allows reducing the random coincidences generated by background photons in the beam on operation. The use of coincidence window as low as 3 ns is reported in the literature for this application [31]. The lines of response (LOR) were formed using the single position information.

The LORs formed a sinogram, which was subsequently analyzed using an ordered subset expectation-maximization (OSEM) method. The sensitivity correction is approximated calculating the system response matrix with a Monte Carlo solid angle-based technique. Scatter and attenuation corrections are not applied [65]. The reconstructed FOV had a volume of 20×12×20 cm^3^ segmented into 1 mm^3^ voxels.

### 2.5. Data Analysis

As stated in Section 1, the key issue in the application of PET as a monitoring system during proton therapy treatment is the reliable reconstruction of the space distribution of the positron emitters produced in the target volume. To address this problem, the data analysis focused on three different aspects of the P&I sensor for PET proton beam monitoring systems, namely the technical specifications of the prototype, the performance of the prototype after beam irradiation and the response of the prototype in the more challenging scenario of beam-on operation.

At first, we verified that the design of a system composed of such a large number of independent LYSO/SiPM sensors had a uniform response and that its performances were controllable and reproducible in the experimental conditions of the proton therapy room. To this aim, the position and width of the energy spectra of each channel collected in the calibration runs at a time distance of 10 h were compared.

Secondly, we analyzed the whole set of PMMA data, including the statistics collected during proton irradiation. We extracted the 2D lateral profile and the 1D profile along the beam direction from the reconstructed image.

The typical longitudinal profile of the reconstructed distribution of the positron-emitter nuclei generated by proton irradiation in the target volume is characterized by a rising and falling edge. We fitted the two edges separately with the function:

(2) $$g\left(z\right)=a_{0}+\frac{a_{1}-a_{0}}{1+\exp\left[a_{2}\left(z-a_{3}\right)\right]}$$

where $a_{0}$ is the background, $a_{1}$ is the maximum value, $a_{2}$ is the fall-off slope and $a_{3}$ is the inflection point. The estimator for the starting point and the ending point of the profile were the values of $a_{3}$ in the two fits. The length of the activity profile was calculated as the difference between the fitted $a_{3}$. The width of the falling edge of the profile was estimated as the inverse of the fall-off slope $a_{2}$.

We investigated the possibility of a precise measurement of the exponential decay of the positron emitters produced in the target volume after proton irradiation, as this is also another key-indicator of the reliability of the P&I sensor for proton therapy monitoring systems. For this purpose, the coincidence rate integrated over the whole FOV was used. A random coincidence subtraction process, based on the delayed window technique, was applied [66]. The average value of 323.5 Hz random coincidence rate was subtracted statistically from the measured rate, using the Poisson counting statistics approximation. The coincidence rate measured in the beam-off state corresponds to the decay of the positron emitters produced in the target volume during proton irradiation. We restricted the analysis to the most abundant elements produced in the experimental conditions of this paper, namely ^15^O, ^11^C and ^10^C. The respective production channels and thresholds are reported in Table 1 following [67].

We fitted the time-dependent coincidence rate with a function consisting of the sum of exponentials:

(3) $$\begin{array}{cc} f\left(t\right)= & A_{\left({}^{15}O\right)}\exp\left[-t/\tau_{\left({}^{15}O\right)}\right]+A_{\left({}^{11}C\right)}\exp\left[-t/\tau_{\left({}^{11}C\right)}\right]+A_{\left({}^{10}C\right)}\exp\left[-t/\tau_{\left({}^{10}C\right)}\right] \end{array}$$

The fit was calculated in the range between $t_{1}=1$ s and $t_{2}=2000$ s after the end of the beam irradiation. The amplitude $A_{i}$ and the half-life $\tau_{i}/\ln2$ of each element were the free parameters of the fit.

The relative abundance $C_{i}$ of each element at the end of the irradiation was calculated from the fit results using the formula:

(4) $$C_{i}=100\times \frac{A_{i}\tau_{i}}{A_{\left({}^{15}O\right)}\tau_{\left({}^{15}O\right)}+A_{\left({}^{11}C\right)}\tau_{\left({}^{11}C\right)}+A_{\left({}^{10}C\right)}\tau_{\left({}^{10}C\right)}}$$

The statistical uncertainty of $C_{i}$ was calculated from the errors of the parameters $A_{i}$ and $\tau_{i}$ estimated in the fit using the standard error propagation technique.

Finally, we investigated the performances of the prototype in the more challenging scenario of the beam-on operation by analyzing the beam-on water phantom data. We studied the specific features of the energy spectra measured in the detector and we extracted the 2D lateral profile and the 1D profile along the beam direction from the reconstructed image. The analysis of the measured profile of the produced positron emitting nuclides is performed using the same technique described above in Equation (2).

To verify whether the measured profiles and nuclide abundance follow the physical expectations, we simulated the interaction of the proton beam with the targets. We used the simulation framework GEANT4, following the suggestions in [29]. The physics list included quark gluon string precompound (QGSP) hadronic model for nucleons, binary ion cascade (BIC) inelastic model for ions and high precision model built upon the TALYS-based evaluated nuclear data library (TENDL) for isotope production. For electromagnetic interactions, we used the available electromagnetic option for medical applications.

A pencil proton beam with energy 150 MeV, energy spread 0.3 MeV and transversal width 2 mm was simulated, which is an approximation of the expected features of the proton beam used in the experiment.

As for the analysis of the space distribution of positron emitters in the target volume, we estimated the length and the falling edge width $\sigma_{phys}$ of the profile of the secondary positron emitters produced in the target volume irradiation using the same fitting technique in Equation (2). The length of the profile was compared directly with the measured value. We interpreted the measured width $\sigma_{meas}$ as the combination of the physical width $\sigma_{phys}$ and of the experimental factors $\sigma_{exp}$, including detector and scanner geometry, reconstruction, random coincidences and noise. The two contributions are independent and they sum up in quadrature:

(5) $$\sigma_{meas}^{2}=\sigma_{phys}^{2}+\sigma_{exp}^{2}$$

The experimental contribution to the width was thus estimated as:

(6) $$\sigma_{exp}=\sqrt{\sigma_{meas}^{2}-\sigma_{phys}^{2}}$$

As for the nuclide analysis, we calculated the relative abundance of the produced ^15^O, ^11^C and ^10^C positron emitters directly storing the physics process information provided by the stepping action during the simulation. The physical expectations obtained in the simulation were compared with the experimental results.

## 3. Results

### 3.1. The Performance of the P&I Sensor System

**Figure 6:**
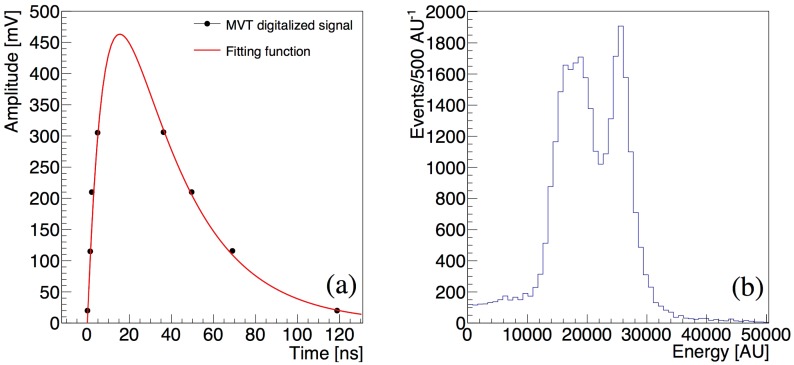
Response of a LYSO/SIPM channel after the detection of a 511 keV photon from a ^22^Na source. In (**a**) the MVT digitized signal (black points) and the fitted function reconstructing the original signal (red line) are shown. The fitted function is integrated within a time window of 200 ns and the distribution of its integral for a small sub-sample of 30,000 events is shown in (**b**): the photoelectric peak and the Compton continuum are visible in the obtained spectrum.

The eight samples generated by the MVT digitizer for a hit pulse in a LYSO/SiPM channel during a ^22^Na calibration run are shown in Figure 6a. The continuous red line in the figure is the estimation of the original signal through the fitting function in Equation (1). The electronic noise introduces a baseline pedestal in the integral spectrum, which is estimated in dedicated noise runs with lowered thresholds. The pedestal-subtracted distribution of a sub-sample of 30,000 events registered in a SiPM/LYSO channel in response to a ^22^Na source during the calibration run is shown in Figure 6b. The spectrum exhibits the typical features of the detection of the 511 keV gamma-ray: a photoelectric peak at approximately 25000 AU and a Compton continuum at lower energies. Since a signal is stored only if all four voltage thresholds are crossed, the energy spectrum exhibits a lower threshold at approximately 14,000 AU.

**Figure 7:**
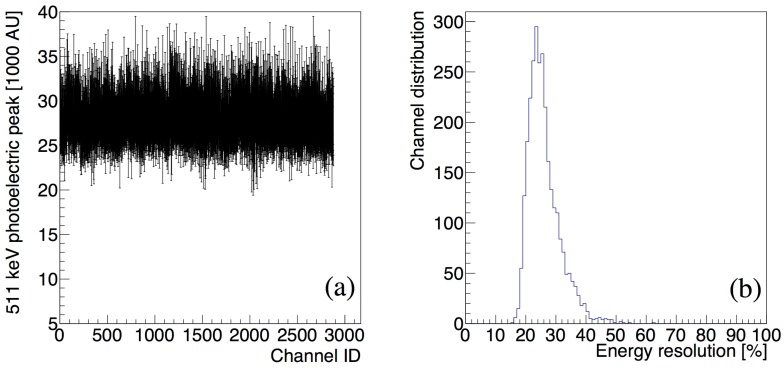
System calibration: position (${\mathrm{ph}}_{\mathrm{pos}}$) (**a**) and energy resolution (FWHM/${\mathrm{ph}}_{\mathrm{pos}}$) (**b**) of the 511 keV photoelectric peak measured during a ^22^Na calibration run in the 2880 channels.

The position of the 511 keV photoelectric peak (${\mathrm{ph}}_{\mathrm{pos}}$) measured during a 10 min ^22^Na calibration run in the 2880 detector channels is shown in Figure 7a. We registered a temperature fluctuation of approximately 1 °C (FWHM) during the acquisition. The error bars represent the width of the photoelectric peak. We observe that the position of the photoelectric peak ranges between 25,000 and 30,000 in all channels. The FWHM of the photoelectric peak is ranging between 5500 AU and 10,000 AU. The distribution of the energy resolution in the 2880 channels composing the proton therapy monitoring PET prototype is reported in Figure 7b. The SiPM/LYSO sensor channels with MVT readout show an average energy resolution (FWHM/${\mathrm{ph}}_{\mathrm{pos}}$) of approximately 24% with a variation (FWHM) of less than 5% across the system.

**Figure 8:**
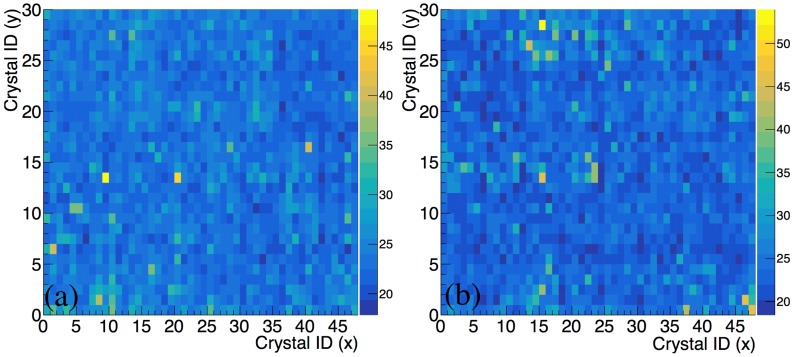
2D histogram of the energy resolution (FWHM/${\mathrm{ph}}_{\mathrm{pos}}$) obtained in the prototype PET monitoring system for the left (**a**) and right (**b**) module with respect to the beam direction.

A 2D histogram of the energy resolution obtained in the two modules of the prototype is shown in Figure 8. We do not observe any sizeable position dependence.

**Figure 9:**
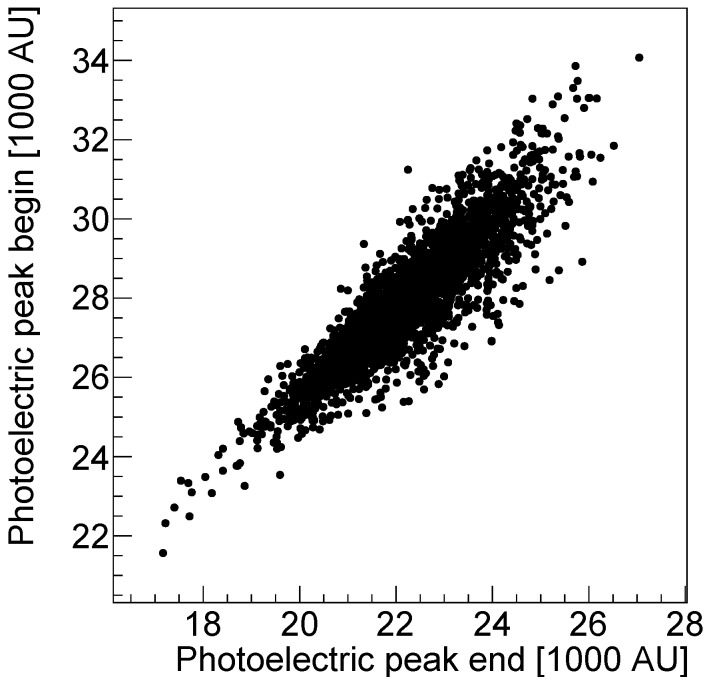
Position of the 511 keV photoelectric peak taken at a time distance of ten hours.

To verify the possibility to control the device in the experimental conditions of the proton therapy room, we plot the position of the 511 keV photoelectric peak taken at a time distance of ten hours in Figure 9. Each data point in the plot corresponds to one of the 2880 independent channels. We registered a temperature increase of approximately 10 °C close to the sensors between the two measurements. The position of the peak exhibits a reduction of approximately 15%. The gain and the photon detection efficiency of the SiPM are in fact decreasing with increasing temperature. We measure temperature variations between different runs up to 25 °C, depending on the working time period of the system.

### 3.2. Lateral Profile of the Positron-Emitting Nuclei

**Figure 10:**
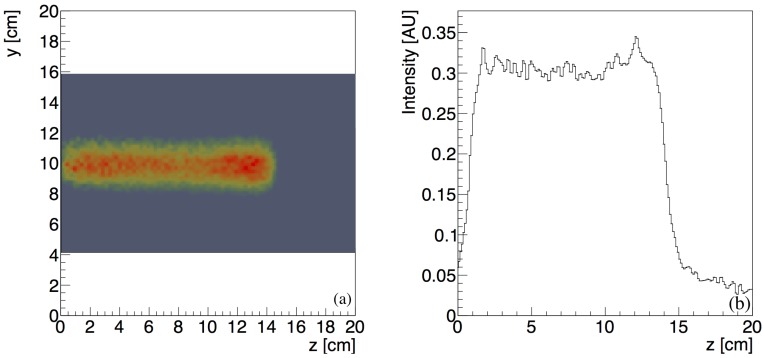
Reconstructed lateral profile at *y* = 10 cm (**a**); and one-dimensional longitudinal profile (**b**) of the positron emitters produced by a 150 MeV proton pencil beam with approximately 2 mm spot-size and 0.3 MeV energy spread within a PMMA target volume.

The reconstructed measured lateral profile of the positron emitters produced in the PMMA phantom after a 150 MeV proton irradiation is shown in Figure 10a. The width of the activity distribution in the y-direction perpendicular to the beam axis reflects the beam spot size, the scattering of the secondary particles produced in the target volume and the intrinsic experimental resolution of the detection system. The gray area corresponds to the reconstructed field of view. The one-dimensional profile of the induced positron activity along the beam *z*-axis at the central position y=10 cm is shown in Figure 10b.

The start-point and end-point of the profile are estimated with the fitting function in Equation (2) as, respectively, $a_{3,s}=\left(0.77\pm 0.32\right)$ cm and $a_{3,e}=\left(14.10\pm 0.23\right)$ cm. The estimated length of the activity profile is $a_{3,e}-a_{3,s}=\left(13.33\pm 0.55\right)$ cm and is in good agreement with the value $\left(13.57\pm 0.02\right)$ cm expected from the simulation.

The starting-edge of the profile has a width of approximately 1 cm due to the acceptance limitations at the borders of the FOV. The Bragg peak appears smeared with a width estimated as $\sigma_{meas}=\left(0.33\pm 0.01\right)$ cm. The width expected from the simulation is $\sigma_{phys}=\left(0.16\pm 0.11\right)$ cm. By comparing the measured and the physical width, following Equation (6), we estimated an experimental contribution of $\sigma_{exp}=\left(2.88\pm 0.03\right)$ mm.

### 3.3. Time Distribution of the Coincidence Rate

The decay half life and the simulated abundance of the ^15^O, ^11^C and ^10^C positron emitters as estimated in the simulation are reported in the second and fourth columns of Table 2, respectively.

**Figure 11:**
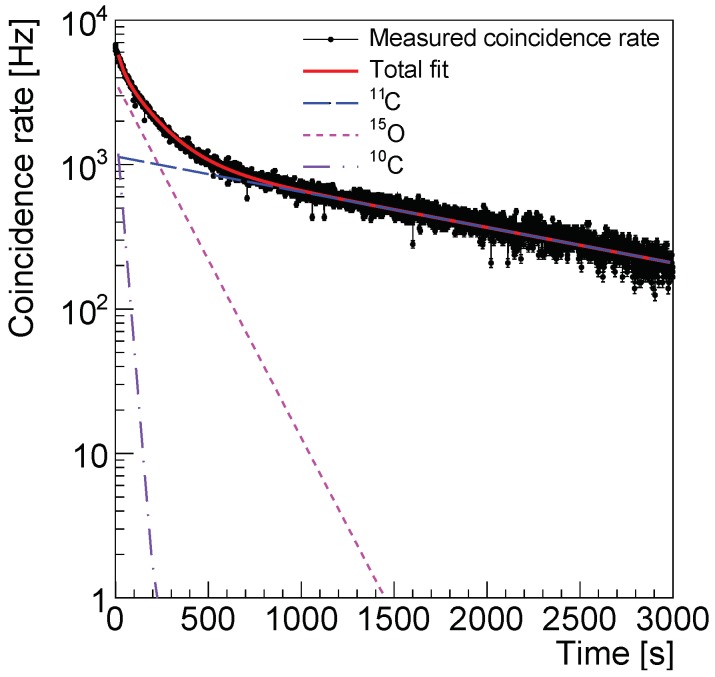
Time dependence of the measured coincidence rate. The exponential decay fitting function and the separate components corresponding to the activated elements are shown.

The measured coincidence rate is shown in Figure 11. The data points were fitted with the function in Equation (3). The total fitting function and each component are displayed in the plot. The fit results are reported in Table 2. The value of the half-life of the three positron emitters was estimated with a relative accuracy of approximately 0.5% and was found in good agreement with the literature values used in the simulation.

The relative abundance of the produced positron emitters was calculated from the fit results using Equation (4) and it is shown in the last column of Table 2. It was estimated with a relative accuracy of approximately 5% and the results were found in good agreement with the expectation from simulation.

### 3.4. Prototype Performance in Beam-On Operation

The signal measured in the prototype after irradiation is generated by the decay of the positron emitting nuclides produced in the target sample. The condition in beam-on operation changes significantly. In fact, a physical background generated by prompt photons arises from nuclear interactions and beam-induced radiation in the experimental area.

**Figure 12:**
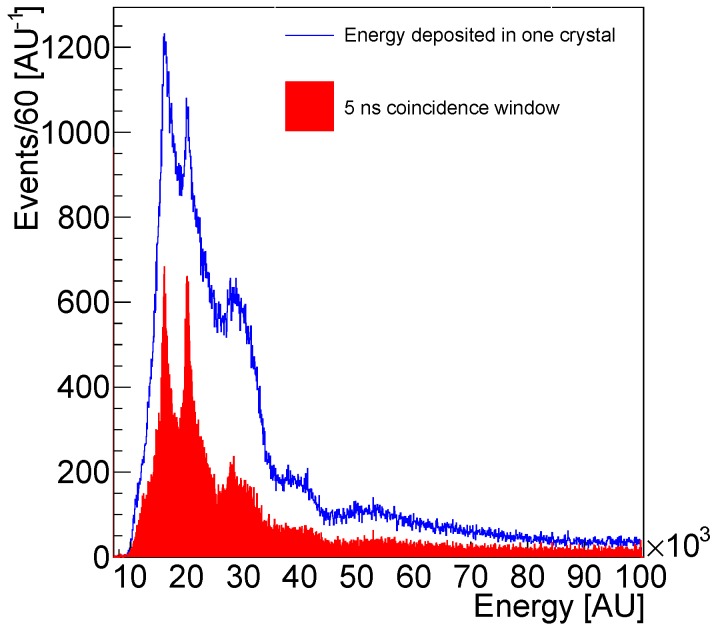
Histogram of the energy deposited in one of the crystals of the PET monitoring system during beam-on operation before (blue line) and after (filled red) 5 ns time coincidence.

The effect of the background is shown in Figure 12 for the case of the water phantom irradiated by a 150 MeV proton beam with irradiation conditions specified in Section 2.3. The blue histogram represents the distribution of the energy measured in one channel of the prototype during beam irradiation without the coincidence time window. The peak corresponding to the photoelectric detection of 511 keV photons is at approximately 20,000 A.U. The Compton component of the spectrum is represented by the peak at approximately 15,000 A.U, due to the sculpting effect of the threshold. In addition, further highly energetic peaks, such as at approximately 30,000 AU, are present in the spectrum, corresponding to proton-beam induced background. The response of the system to high energies is not linear and it is not possible to estimate with precision the energy correspondent to the highly energetic peaks. The red histogram represents the energy distribution of the events after time coincidence selection. We observe that most of the beam-induced background is removed when only signals are taken from one of the crystals, which are in 5 ns time coincidence with any other crystal in the opposite panel. The photoelectric peak and the Compton structure are well visible after event selection.

**Figure 13:**
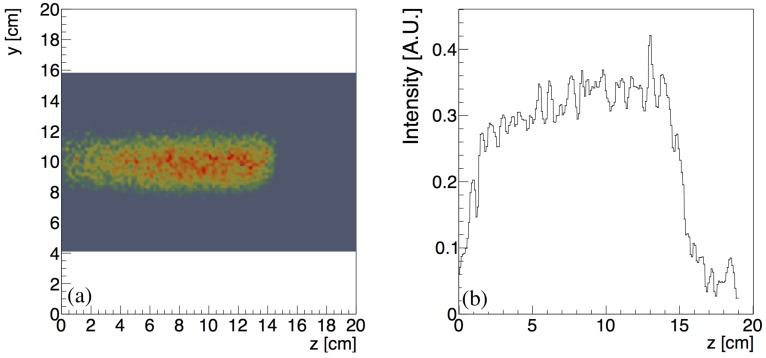
Reconstructed lateral profile at at *y* = 10 cm (**a**); and one-dimensional longitudinal profile (**b**) of the positron emitters produced by a 150 MeV proton pencil beam with approximately 2 mm spot-size and 0.3 MeV energy spread within a water target volume during beam-on operation.

The reconstructed measured lateral profile of the positron emitters produced in the water phantom during a 150 MeV proton irradiation is shown in Figure 13a. The width of the activity distribution in the *y*-direction perpendicular to the beam axis reflects the beam spot size, the scattering of the secondary particles produced in the target volume and the intrinsic experimental resolution of the detection system. The gray area corresponds to the reconstructed field of view. The one-dimensional profile of the induced positron activity along the beam *z*-axis at the central position y=10 cm is shown in Figure 13b.

The start-point and end-point of the profile are estimated with the fitting function in Equation (2) as, respectively, $a_{3,s}=\left(0.80\pm 0.21\right)$ cm and $a_{3,e}=\left(14.22\pm 0.30\right)$ cm. The estimated length of the activity profile is $a_{3,e}-a_{3,s}=\left(13.42\pm 0.51\right)$ cm and is in good agreement with the value $\left(13.30\pm 0.02\right)$ cm expected from the simulation.

## 4. Discussion

We tested a prototype of PET monitoring system for proton therapy obtained with the P&I sensor based on the LYSO/SiPM optical sensors. The size of the prototype compares well with other systems developed in previous studies. Panel PET prototypes with size ranging between 6×6 cm^2^ and 10×25 cm^2^ are reported in the literature [30,31,36,37,38]. In line with the most recent advances of this technique, we performed the experiment in-beam, during both beam-on and beam-off time.

The key improvement obtained in this paper is the unique digital readout scheme applied to the LYSO/SiPM sensor system. The MVT technique proposed here offers a possible alternative both to the constant 1 GHz sampling [68,69,70,71,72,73,74] and to the single threshold approach [38]. All PET monitoring systems based on scintillator/SiPM sensing technology suffer from intrinsic limitations in count rate determined by the proper decay time of the chosen scintillator. However, the digitization stage of the readout electronics usually imposes the most stringent limitation to the maximal allowed count rate. The novel sensor readout configuration specifically designed for SiPM-based systems allowed reaching a maximal count rate ranging between 0.25 Mcps [36] and 3.5 Mcps [38], due to limitations in readout architecture and data transfer solutions. The count rate limitation of a PET monitoring system based on one of the most advanced SiPM (PHILIPS) is reported to be 1.3 Mcps [75]. The saturation count rate obtained in this study during the beam-on time is 5.2 Mcps and overwhelms the above limits significantly. The imaging of proton induced activity in the beam-on condition is possible with the P&I sensor system.

The high count rate is obtained reducing the sampling frequency. Clearly, having only eight samples available, the digital signal processing capabilities and analysis techniques are limited with respect to the 1 GHz sampling frequency technology [68]. This has an impact on the energy resolution of the 511 keV photoelectric peak. We measured an energy resolution of approximately 23%, against the 12–14% obtained in other PET monitoring systems based on scintillator/SiPM sensing technology with different readout electronics [30,31,36,37,38]. This result indicates that a better fitting strategy than the one proposed in Equation (1) needs to be investigated to improve the energy resolution of the PET monitoring system.

Besides the optimization of the fitting strategy, we found that a better cooling of the system is needed. Both the detector heads and the MVT boards have sizeable temperature fluctuations. SiPM sensors are based on the impact-ionization process, the probability of which decreases with increasing temperature. Thus, a higher internal electric field is needed in the SiPM structures for higher temperature. When the temperature has a positive change and the bias voltage is kept fixed, the breakdown voltage of the SiPM sensors increases and the gain and the photon detection efficiency of the SiPM sensors decreases. As a result, the position of the 511 keV photoelectric peak has a negative shift when the temperature increases. The changes in the gain during the data acquisition deteriorates the energy resolution of the detector. This is a common feature of all SiPM-based compact systems and can be controlled with periodic calibration runs, temperature correction of the calibration constants, as performed for example in [76] and with a dedicated cooling system as in [36].

Moreover, the four voltage thresholds need to be adjusted according to the changing signal amplitude. Currently, they are fixed according to a characterization performed in the laboratory environment in the production stage, which is optimal for the operation of traditional Positron Emission Tomography systems with controlled temperature. The large energy resolution measured in the prototype suggests that an optimization of the voltage sampling thresholds is needed to adapt the sampling to the real-time amplitude of the LYSO/SiPM signals.

Another effect to be further investigated is the deterioration of the tensile strength of the optical glue, due to both local temperature changes above allowed temperature limits and exceeding shear stress. Consequently, the optical coupling between crystals and SiPMs deteriorates and the energy resolution is subject to degradation. The results suggest a verification of the used optical glue and a comparison with other available glues.

The LYSO/SiPM sensing channels constituting the P&I system exhibit a non-uniformity of less than 5% in both position and width of the 511 keV photoelectric peak. This result shows that the SiPM technology is mature for the construction of large scale systems, as already noted in [37], where a 10% non-uniformity of a LYSO/SiPM-based PET system for proton therapy monitoring is reported.

As for the longitudinal profile of the reconstructed positron emitters activity in the target volume after irradiation, we obtained a spatial resolution of approximately 3 mm (FWHM), which is consistent with the size of the pixels of the prototype. This accuracy is following the improvement of the performances for PET monitoring systems provided with the SiPM. However, SiPM-based PET monitor systems exhibited better values, ranging between 1.5 mm and 3 mm [30,31]. This suggests that the cross-sectional size of the LYSO/SiPM detection system can be decreased to improve the spatial accuracy. Besides these hardware considerations, the measured longitudinal length of the positron emitters distribution in the target volume was consistent with the simulation expectations. However, a proper comparison between the features of the profiles in measurement and in simulation requires introducing in the simulation the geometry of the detector, the properties of the electronic chain for the generation of the electric signals and the reconstruction of the simulated digitized hits [67].

Finally, the time stamping obtained with the MVT method applied to the LYSO/SiPM detection system allows reconstructing both the decay and the relative abundance of the positron-emitters ^15^O, ^11^C and ^10^C produced in the phantom with the irradiation of $10^{10}$ protons in approximately 15 s with relative accuracies of 0.5% and 5%, respectively. Our results are in agreement with previous studies [7,13,14,19,31]. While maximal coincidence rates ranging between 100 Hz and 2 kHz are reported in the nuclide analysis of the decay, we observe a maximal measured coincidence rate of 7 kHz 1 s after the beginning of the beam-off time. The larger measured coincidence rate statistics is a direct consequence of the higher count rate capability of the P&I sensor system presented in this paper with respect to other prototypes. Recent studies have shown that SiPM-based PET monitoring systems can resolve also positron emitters with shorter half life, as ^8^B (0.7 s) [37] and ^12^N (11 ms) [36], in the inter-spill times during proton irradiation. The accuracy of the results obtained in this paper and the larger collected statistics suggests that the immediate future direction of our study is a dedicated experiment for the identification and reconstruction of fast-decaying positron emitters produced in the target volume during proton irradiation.

## 5. Conclusions

In the present study, we measured the performance of a the P&I sensor technology based on LYSO/SiPM systems with digital MVT readout used in a PET monitoring system for proton therapy both in beam-on and in beam-off operational conditions. The key property of the P&I concept is the higher maximal allowed count rate with respect to other available sensing technologies. The spatial resolution of the prototype is approximately 3 mm (FWHM). The count rate capability of the MVT electronics guarantees collecting good statistics for precise estimation of the decay time and relative abundance of the produced positron emitters with accuracies of approximately 0.5% and 5%, respectively, after irradiation with $10^{10}$ proton within approximately 15 s.

The results show that the P&I sensor is mature for application to non standard PET systems for the monitoring of the delivered dose during proton therapy treatment. A drawback of the sensors is the relatively large space occupied by the digital readout boards. We are currently developing new SiPM sensors in CMOS technology that will allow integrating most of the MVT electronics on the chip, thus improving the compactness of the system design.

## Figures and Tables

**Table 1:** Most relevant positron emitters produced in the target volume and production channels used in this study.

Isotope	Channel	Threshold [MeV]
^15^O	^16^O(p,pn)^15^O	16.79
^11^C	^12^C(p,pn)^11^C	20.61
	^16^O(p,3p3n)^11^C	59.64
^10^C	^12^C(p,p2n)^10^C	35
	^16^O(p,3p4n)^10^C	72

**Table 2:** Relative abundance and half life of the isotopes produced after proton irradiation in the Monte Carlo simulation and experimental results after fitting the measured coincidence rate.

Element	Half life Literature [s]	Half Life Measurement [s]	Abundance Simulation (%)	Abundance Measurement (%)
^15^O	122	121.8±0.6	27± 2	25±3
^11^C	1220	1218±6	70±3	73±2
^10^C	19.3	19.13±0.09	3.0±0.2	2.6±0.2

## Abbreviations

The following abbreviations are used in this manuscript:

PET	Positron Emission Tomography
P&I	Plug&Imaging
DCR	Dark Count Rate
SiPM	Silicon Photomultiplier
CMOS	Complementary Metal Oxide Semiconductor
LYSO	Lutetium Yttrium Orthosilicate
MVT	Multi Voltage Threshold
TDC	Time to Digital Converter
LVDS	Low Voltage Differential Signaling
FPGA	Field Programmable Gate Array
FOV	Field Of View
PMMA	Polymethylmethacrylat
AU	Arbitrary Units
OSEM	Ordered subset expectation maximization
